# The Relationship and Changes of Liver Blood Supply, Portal Pressure Gradient, and Liver Volume following TIPS in Cirrhosis

**DOI:** 10.1155/2022/7476477

**Published:** 2022-12-08

**Authors:** Jinghong He, Jinyu Li, Changxing Fang, Ying Qiao, Duiping Feng

**Affiliations:** ^1^Department of Radiology, First Hospital of Shanxi Medical University, Taiyuan 030001, Shanxi, China; ^2^Department of Oncological and Vascular Intervention, First Hospital of Shanxi Medical University, Taiyuan 030001, Shanxi, China

## Abstract

**Aim:**

Transjugular intrahepatic portosystemic shunt (TIPS) alters the liver blood supply and reduces portal pressure. This study was to investigate the changes and associations of the hepatic blood flow, liver volume, and portal pressure gradient (PPG) after TIPS in liver cirrhosis.

**Methods:**

Twenty-one patients with liver cirrhosis who received TIPS were recruited. The contrast CT images were used to assess the iodine density (ID) of liver parenchymal and liver volume. The ID of the liver parenchyma was used to reflect hepatic blood flow. We used a paired *t*-test and regression analysis to investigate the effect of TIPS on hepatic blood flow, liver volume, and PPG in individuals with cirrhosis and the factors that affect changes in liver volume.

**Results:**

After TIPS, there was a significant improvement in the ID of liver parenchyma at arterial phase (AP) and PPG in individuals with cirrhosis (*P* < 0.05). Each 1 unit increase in the ID change of whole liver parenchyma at the venous phase (VP) was significantly associated with a 269.44 cm^3^ increase in the liver volume after TIPS (*b* = 269.44, *P* = 0.012). With an increasing ID change of whole liver parenchyma at VP, the change in liver volume followed an increasing trend (*P* for overall association = 0.005).

**Conclusions:**

Our data indicate that there was a significant improvement in hepatic blood flow, especially at AP, after TIPS and the change in hepatic blood supply from the portal vein is positively associated with the change in liver volume after TIPS. Increasing the blood supply to the liver from the portal vein may improve the reduction of liver volume.

## 1. Introduction

Liver cirrhosis is a common chronic gastrointestinal disease in China [1], which seriously affects the survival rate and quality of life [2]. Cirrhosis makes it hard for blood to flow in the portal venous system [3]. This resistance creates a backup of blood and increases pressure. This results in portal hypertension. Portal hypertension is a major complication of cirrhosis, and its consequences, including ascites, esophageal varices, hepatic encephalopathy, and hepatorenal syndrome, lead to substantial morbidity and mortality [4]. Transjugular intrahepatic portosystemic shunt (TIPS) is an effective treatment for portal hypertension and its complications in cirrhotic patients [5]. This shunt diverts blood flow from the portal system directly into the systemic circulation, reducing portal pressure and altering blood supply to the liver, thereby helping resolve ascites and reducing the risk for variceal hemorrhage [6]. We speculate blood supply may be decreased in liver parenchyma after TIPS since portal vein blood partially flows directly to the inferior vena cava through a portal-systemic shunt. It is of great significance to clarify the blood supply changes of liver parenchyma after TIPS for evaluating liver volume changes, guiding the next treatment, and preventing liver failure.

The iodine density (ID) of liver parenchymal with spectral CT has been widely used as a measure of hepatic blood supply [7, 8]. A study reported that the ID of hepatic parenchyma in the arterial phase (AP) increased after TIPS [8]. But which part of the liver's blood supply changed has not been explored. Besides, the effect of TIPS on hepatic blood supply and liver volume in individuals with liver cirrhosis is not clear. No studies have clearly indicated the factors causing liver volume changes induced by TIPS.

Therefore, this study compares the ID of liver parenchyma, liver volume, and portal pressure of individuals with liver cirrhosis before and after TIPS and explores their possible association.

## 2. Materials and Methods

### 2.1. Study Population

Medical records and video material were retrospectively reviewed for 21 patients who had TIPS procedures for liver cirrhosis from 2019 to 2020. The inclusion criteria were as follows: (1) patients with liver cirrhosis; (2) contrast CT was performed within 1 week before and 2-3 months after TIPS; and (3) indications for TIPS insertion were refractory ascites and secondary prevention of variceal bleeding. The exclusive criteria were as follows: (1) patients with malignant hepatic tumors, either primary or metastatic; (2) any conditions affecting liver blood flow; (3) patients with allergies to iodinated contrast media; and (4) other factors affecting contrast CT examination. The study was approved by the Research Ethics Committee of the First Hospital of Shanxi Medical University.

### 2.2. Spectral CT Examination and Quantitative Indices Measurement

The selected images are collected from spectral multi-slice spiral CT (IQon spectral CT, Philips Healthcare, Cleveland, OH, USA). The tube voltage is 120 kV, the tube current adopts automatic tube current control technology, the rotation speed of the rack is 0.5 s/cycle, the width of the collimator is 64 mm *∗* 0.625 mm, the pitch is 0.929, and the contrast media was ioversol (Jiangsu Hengrui Pharmaceuticals Co., Ltd; specification: 74.1 g/100 ml). The contrast media injection rate is 3.0 mL/s (1.5 mL/kg). The contrast agent intelligent tracking threshold triggering technology is used in the enhanced scanning at AP. The region of interest (ROI) was set in the abdominal aorta, with a threshold of 110 HU, and the venous phase (VP) scan was started 35 s after the end of the AP. The obtained data were reconstructed by spectral reconstruction to generate a spectral-based image, and then the spectral-based image was imported into the Philips SpDS image workstation (Spectral Diagnostic Suite 6.5, Philips Healthcare) for analysis to obtain an iodine concentration image.

### 2.3. Iodine Density and Liver Volume Measurement

The ID and liver volume measurement refer to previous reports [8]. In brief, the ID was measured on iodine-based material decomposition images, including noncontrast, AP, and VP phases. Multiple ROIs were placed in liver parenchyma from different hepatic lobes. The ID of the portal vein and abdominal aorta were also measured. Liver volume was measured on images of an enhanced CT at VP. The images were analyzed by software named as total liver and segment separation in Workstation (SiemensSyngo.via), then “region growing” was applied, and finally the liver volume would be calculated automatically and shown with volume rendering.

### 2.4. Statistical Analysis

All analyses were completed with SPSS software (version 24.0) and SAS software (version 9.4), as previously reported [9]. Continuous variables are expressed as mean ± standard deviation or median (interquartile range). Categorical variables are presented as numbers (proportion). Before analysis, normality tests or *P* − *P* plots were used to test the normality of the variables. For normal-distribution variables, a paired *t*-test was applied to explore changes from baseline. For variables that were not normally distributed, Wilcoxon signed-rank was applied. A Spearman correlation analysis was used to evaluate the association between the change in ID, portal pressure, and liver volume before and after TIPS. Then multiple linear regression analysis by step method was used to investigate the effect factors of the change of liver volume after TIPS. For models with significant statistical association estimates, we further used restricted cubic spline models to assess the shapes of the dose-response associations. In this study, all “D_” refers to the value of a variable after TIPS minus the value of this variable before TIPS. In all cases, bilateral *P* values less than 0.05 were considered statistically significant.

## 3. Results

Table 1 shows the baseline characteristics of the 21 study participants. Age ranged from 42 to 74 y, with a mean of 55.0 y. The number of men was lower than the number of women in the population (47.6% and 52.4%, respectively). The etiology of liver cirrhosis were mainly hepatitis virus (42.9%) and alcohol (33.3%). The median follow-up time was 55 days.

Pre- and post-TIPS, the ID of liver parenchyma, portal pressure gradient (PPG), and liver volume are summarized in Table 2. The ID of liver parenchyma at AP was significantly increased after TIPS, including left lobe of liver, right lobe of liver and whole liver (*P* < 0.05). By contrast, there was no statistically significant change in the ID of liver parenchyma in VP after TIPS. For portal vein, the ID at AP were increased (*P* < 0.05), while ID at VP was stable after TIPS. However, the ID in the aorta was decreased after TIPS (11.48 ± 3.28 vs. 9.64 ± 2.08, *P* = 0.013). There was improvement in PPG after TIPS (28.72 vs. 20.08, *P* < 0.001). Liver volume was reduced after TIPS (976.85 cm^3^ vs. 862.35 cm^3^, *P* = 0.010).

**Figure 1:**
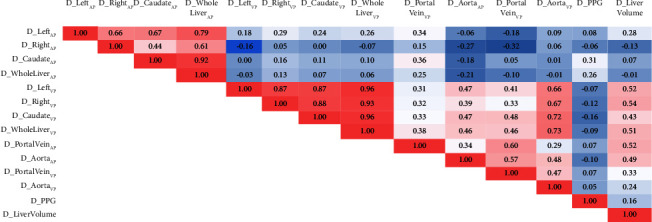
The Spearman correlation analysis of the change in ID, PPG, and liver volume pre-andpost-TIPS. AP: arterial phase; VP: venous phase; PPG: portal pressure gradient. D_ represented the difference in iodine density before and after TIPS; the bold presented the *P* value less than 0.05.

Spearman correlation analysis investigated the association between change in ID, PPG, and liver volume before and after TIPS. The results showed that the ID values of different parts of the liver parenchyma were positively correlated in the AP, and the ID values in the VP were positively correlated with each other. The ID of the aorta was positively correlated with the ID of the hepatic parenchyma in the VP. No correlation was found between PPG and ID values. The change in liver volume was positively correlated with the ID of hepatic parenchyma at VP, portal vein, and aorta at AP (*P* < 0.05) (Figure 1).

**Figure 2:**
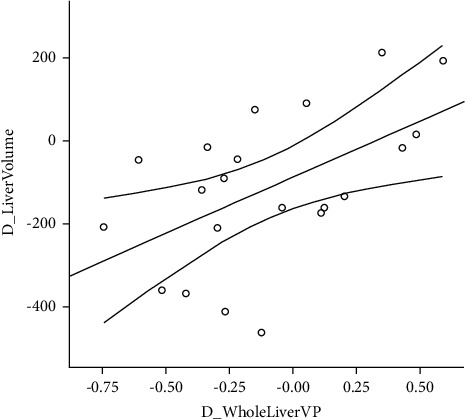
Scatter plots between the change of whole liver parenchyma iodine density at venous phase (D_WholeLiver VP) and the change of liver volume (D_LiverVolume).

**Figure 3:**
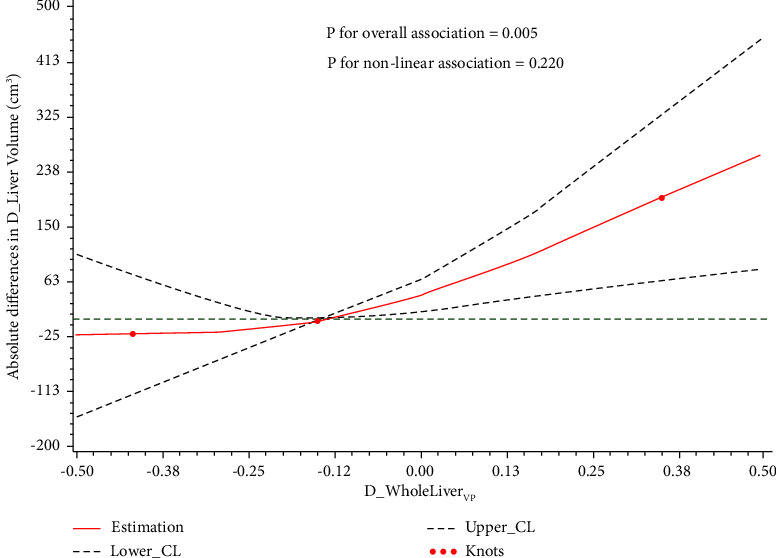
Restricted cubic spline models of association of the change of whole liver parenchyma iodine density at venous phase (D_WholeLiver_VP_) with the change of liver volume (D_LiverVolume). The reference is the median of D_WholeLiver_VP_ levels; dashed lines represent 95% confidence intervals; and red knots represent D_WholeLiver_VP_ at the 15th, 50th, and 85th percentiles. D_WholeLiver_VP_: the change of whole liver parenchyma iodine density at venous phase; D_LiverVolume: the change of liver volume.

The scatter plots showed a linear association between the change of liver volume and the change of whole liver parenchyma iodine density at the venous phase (D_WholeLiver VP), indicating that 28.8% (*R*^2^ = 28.8) of the overall variation depends on D_WholeLiver VP (Figure 2). Then multiple linear regression by the step method showed that D_WholeLiver VP was significantly and positively associated with the change liver volume (*b* = 269.44, *P* = 0.012) (Table 3). To improve the relationship, we further used restricted cubic spline models to elevate the dose-response relationship between D_WholeLiver VP and D_LiverVolume. Restricted cubic spline models showed that with increasing D_WholeLiver VP, D_LiverVolume had an increasing trend (*P* for overall association <0.05) (Figure 3).

## 4. Discussion

To our knowledge, this is the first time to investigate the relationship between hepatic blood flow changes and liver volume changes after TIPS. At the same time, we describe the changes in hepatic blood flow and liver volume after TIPS. We observed that there was a significant improvement in hepatic blood flow, especially at AP, after TIPS. We also found that the change in the ID of whole liver parenchyma at VP was positively associated with the change in liver volume.

TIPS is an effective treatment for portal hypertension with esophageal varices and ascites [10]. A portosystemic shunt after TIPS can change the hepatic blood flow, which further induces some complications such as hepatic encephalopathy [5, 11]. Assessment of liver blood flow change after TIPS is of great value for preventing complications and reducing the risk of liver failure. The liver receives a dual blood supply from the hepatic portal vein (75%) and hepatic arteries (25%) [12]. This study found that the ID of the hepatic parenchyma increased in the AP and decreased in the PV, indicating that the blood supply to the liver from the hepatic artery increases and the blood supply from the portal vein to the liver is reduced. Walser et al. reported that the mean arterial contribution to liver blood flow was 25.4% in the normal control patients, 39.9% in patients prior to TIPS, and 48.3% after TIPS [13]. Preibsch et al. also found that following TIPS placement, arterial liver perfusion and, additionally, the hepatic perfusion index increased in all patients, whereas portal venous liver perfusion markedly decreased, whereas total perfusion remained unchanged [14]. This is consistent with our conclusion that the hepatic blood supply is redistributed after TIPS. Shunts can cause partial portal vein blood to flow directly back into systemic circulation, so that portal vein blood supply to the liver parenchyma is further reduced. There was also a statistically significant decrease in PPG after TIPS in this study, which was consistent with previous studies [15, 16].

The present study also found a statistically significant reduction in liver volume within 2-3 months after TIPS. A previous study reported that the average loss of liver volume was 0.74 mL per day after TIPS, which was associated with higher bilirubin levels [17]. We speculate that there are two reasons for the reduction of liver volume after TIPS. First, the process of liver cirrhosis is not inhibited, and the liver continues to shrink. Second, the blood supply to the liver was altered after TIPS. The decrease in portal venous blood flow was accompanied by compensatory increases in the hepatic arterial blood supply, as confirmed in these results. The normal blood supply to the liver comes from 75% of the portal vein and 25% from the hepatic artery [13]. The reduction of portal vein blood supply may not be fully compensated by the hepatic artery, resulting in a decrease in total hepatic perfusion and a reduction in liver volume. We are more inclined to the second speculation, but related research is needed to evaluate the changes in the overall blood supply of the liver after TIPS, and relevant studies suggest that liver perfusion decreases after TIPS [18]. This also confirms our hypothesis. As the shunt is established, liver volume may change over time. However, a study reported that total liver perfusion remained unchanged after TIPS [14], and we speculate that this may be related to the follow-up time. After the shunt channel is established, the body will gradually compensate by changing the blood supply to the liver.

In addition, in this study, we also explored the factors that affect the change in liver volume after TIPS, and the regression results showed that the ID of the whole liver parenchyma at the VP was positively correlated with liver volume. The changes in ID in liver parenchyma in the VP indicate that the liver volume is more affected by the hepatic portal blood supply and indirectly reflect that TIPS may not reverse liver cirrhosis in the short term, at least in the next 2-3 months. TIPS can decrease portal hypertension, but it was originally considered a bridge therapy before liver transplantation in patients with cirrhosis [19].

Some limitations must be acknowledged in the present study. Firstly, this study has a rather small sample. But, our results also provide some data about changes in liver blood supply and liver volume after TIPS and their relationship. The large multicenter studies should be performed to confirm our conclusions. Secondly, in this study, contrast-enhanced CT is performed at two time points, including arterial and venous phases. Finally, we used indicators that indirectly reflect hepatic blood supply in the liver parenchyma. But the ID has been proven in relevant studies to reflect liver blood supply, and it is an economical and convenient indicator.

## Figures and Tables

**Table 1:** Participant characteristics (*n* = 21).

Characteristic	Value
Age (year)	55.0 ± 8.8
Gender (men)	10 (47.6%)
Etiology of liver disease	
Hepatitis virus	9 (42.9%)
Alcohol	7 (33.3%)
Other	5 (23.8%)
Follow-up (days)^a^	55.0 (48.0, 95.0)

Values are expressed as mean ± SD or number (%). ^a^Median with interquartile range.

**Table 2:** Comparisons of iodine density, portal pressure gradient, and liver volume before and after TIPS.

Items	Pre-TIPS	Post-TIPS	*P* value
Hepatic parenchyma			
Left lobe of liver (mg/ml)			
AP	0.26 ± 0.18	0.42 ± 0.20	**0.001**
VP	1.51 ± 0.34	1.47 ± 0.32	0.647
Right lobe of liver (mg/ml)			
AP	0.21 ± 0.16	0.34 ± 0.23	**0.011**
VP	1.46 ± 0.34	1.40 ± 0.29	0.408
Caudate lobe (mg/ml)			
AP	0.35 ± 0.19	0.49 ± 0.31	0.066
VP	1.72 ± 0.28	1.54 ± 0.43	0.073
Whole liver (mg/ml)			
AP	0.27 ± 0.16	0.41 ± 0.23	**0.006**
VP	1.56 ± 0.30	1.48 ± 0.33	0.377
Portal vein (mg/ml)			
AP	1.55 ± 0.90	3.85 ± 1.86	**<0.001**
VP	4.48 ± 1.26	5.92 ± 2.69	0.073
Aorta (mg/ml)			
AP	11.48 ± 3.28	9.64 ± 2.08	**0.013**
VP	4.06 ± 0.67	4.16 ± 0.86	0.652
PPG (mmHg)	28.72 ± 2.99	20.08 ± 3.70	**<0.001**
Liver volume (cm^3^)	976.85 ± 325.33	862.35 ± 307.09	**0.010**

AP: arterial phase; VP: venous phase; PPG: portal pressure gradient; the bold presented the *P* value less than 0.05.

**Table 3:** Linear regression analysis by the step method to explore the risk factors of the change in liver volume before and after TIPS.

D_LiverVolume	*B*	95% CI	*P*
D_WholeLiver_VP_	269.44	66.06–472.83	**0.012**

D_LiverVolume: the change in liver volume before and after TIPS; D_WholeLiver_VP_: the change iodine density of whole liver venous phase before and after TIPS; the bold presented the *P* value less than 0.05.

## Data Availability

The datasets used and analyzed during the current study are available from the corresponding author on request.

## Abbreviations

TIPS: Transjugular intrahepatic portosystemic shunt
ID: Iodine density
PPG: Portal pressure gradient
AP: Arterial phase
VP: Venous phase
ROI: Region of interest.
